# Linking basic principles of solution chemistry to kidney stone formation timelines

**DOI:** 10.1038/s41598-025-07193-1

**Published:** 2025-07-01

**Authors:** Ishai Dror, Claude Merlin, Yaniv Shilo, Brian Berkowitz

**Affiliations:** 1 Department of Earth and Planetary Sciences, Weizmann Institute of Science, Rehovot, 7610001 Israel; 2 Department of Urology, Kaplan Medical Center, Affiliated with the Hebrew University, Rehovot, 7661041 Israel

**Keywords:** Stone formation, Stone growth rate, Supersaturation, Calcium oxalate, Calcium phosphate, Uric acid, Biochemistry, Urology

## Abstract

Kidney stone formation remains enigmatic, largely because of its multifactorial nature, and because assessment is by necessity based mostly on *a posteriori* analysis and/or on specific analyses (e.g., prescribed components found in urine) that focus on particular conditions. Here, we offer a different perspective to assess overall aspects of stone formation, delineating a method to calculate minimum times of stone formation as a function of stone type (calcium oxalate, calcium phosphate, and uric acid) and size (up to 10 mm), in the form of the pure mass of the stone, without consideration of actual formation or aggregation causes or processes. The calculations thus represent characteristic measures that delineate limits on times required to form specific stone volumes as a function of chemical content and mass. The times to form each stone type and specific size vary considerably, ranging from days to years. A key factor is the amount of the “building block” material in urine that actually contributes to stone formation, i.e., the % yield from solution to solid phase. In some cases, unrealistically high yields (e.g., 5–10%) are required to form a stone with a specified size, type, and time; this indicates that other factors – at least some of which can be deduced from the analysis – play key roles in stone formation. This information thus provides estimates that constrain assessments of stone formation mechanisms and interpretation of clinical findings.

**Supplementary Information:**

The online version contains supplementary material available at 10.1038/s41598-025-07193-1.

## Introduction

How long does it take a kidney stone to form? And what are the underlying controls on stone formation? Decades of research have focused on efforts to understand and quantify kidney stone formation, and yet there remain enormous knowledge gaps in terms of rigorous measurements and delineation of an authoritative mechanistic understanding of kidney stone formation and growth rates.

There are two fundamental “unknowns”, particularly with regard to calcium oxalate, calcium phosphate, and uric acid stones (here, we exclude infectious (struvite) or genetic (cystine) etiologies). First, under what conditions do calcium oxalate, calcium phosphate, and uric acid precipitate as small (~ 10–20 μm average diameter) crystals^1–3^, and subsequently combine to form aggregates as calcium oxalate/phosphate and uric acid stones? Second, what are the typical growth rates of stones until they reach sizes of particular clinical relevance (diameters of ~ 3–5 mm)? While stone recurrence following natural expulsion or endourological intervention ranges from ~ 10–60% of patients and generally occurs after 5, 10, or more years^4–6^, stone recurrence is anecdotally reported to occur in some patients as quickly as several weeks or a few months. While one might thus conclude that stones of clinical significance can form within weeks or over many years, it must be recognized that these are reported times from a *previous* stone occurrence to the *recurrence* of pain and clinical identification. Other studies report stone formation times ranging up to two decades, based on, e.g., atmospheric bomb pulse dating^7^ and analysis of lifestyle events, clinical data, and urinary stone events^8^. Ultimately, the actual time required for stone formation within a kidney may be relatively short, but the presence of the stone may not present as a clinical pathology until its release to the ureteropelvic junction or ureter at some later time.

We emphasize here that the terminology “stone precipitation” used in some literature is misleading: kidney stones do not precipitate as large single crystals, nor do small crystals “precipitate” to form a large stone. Rather, kidney stones actually consist of complex, structurally heterogeneous aggregates of small, previously-formed crystals, and possibly with other existing particles, that together form crystalline-like solids. In other words, large kidney stones do not simply “precipitate”; rather, they “form” as composite crystalline-based objects. The often layered (cross-sectional) structure of such stones suggests that the aggregation process for stone formation may occur in multiple steps over time, rather than as a single aggregation event^9–11^. So key questions remain: what controls the aggregation of small crystals, and what is the rate of this aggregation process in kidney stone formation? The answers to both questions remain highly enigmatic, but we can gain insights into the latter one by considering a purely chemical approach.

This study offers a different perspective to address the rate of stone formation, by developing and applying a method to calculate minimum stone formation times as a function of prescribed stone type and size. The calculations here are based purely on a chemical accounting of elements present in urine – as reported in urine chemistry analysis – and in the stone itself, to assess the volume and mass of a stone as a function of time, without specific consideration of the properties of small crystals comprising the stone or of the (abiotic and biotic) processes of their precipitation and aggregation. The calculations thus represent characteristic measures that delineate limits on times required to form specific stone volumes; this information helps to constrain assessments of stone formation mechanisms and interpretation of clinical findings.

## Methods to quantify kidney stone formation

### Precipitation of single crystals

Kidney stones (calcium oxalate, calcium phosphate, and uric acid) do not appear as large (mm or even cm scale), “uniform” or “homogeneous” crystal structures. This is evident, for example, by literature reporting stone surfaces and cross-sectional cuts by optical and scanning electron microscopy (SEM)^10,11^. Indeed, SEM images indicate disordered aggregates of small (~ 1–20 μm) crystals, often in distinct layers wherein each layer comprises a disordered aggregate of smaller crystals^12^. In some cases, kidney stones then develop as aggregates of these larger layered units, being bound together and containing additional enclosing deposition layers.

In controlled in vitro settings, calcium oxalate, calcium phosphate, and uric acid can be precipitated as crystals with diameters/lengths of the order of 1–100 μm. More specifically: calcium oxalate monohydrate (whewellite) and dihydrate (weddellite) do not generally precipitate as crystals larger than about 1–15 μm in diameter^13,14^; calcium phosphate crystals (often classified as either brushite or hydroxyapatite) form a range of shapes (~ 5–100 μm) as well as smaller plate-like crystals (~ 1–3 μm)^15,16^, while uric acid crystals tend to form as thin elongated crystals (up to ~ 100 μm length) and agglomerated plate-like crystals (~ 20–100 μm)^17^. Interestingly, similar sizes are reported in studies of crystals sampled from urine^1–3^.

### Estimating the stone mass and time to stone formation

We examine – based on purely geometrical and chemical considerations – the growth of pure mineral in the forms of (i) calcium oxalate monohydrate (whewellite), (ii) calcium oxalate dihydrate (weddellite), (iii) hydroxyapatite, (iv) brushite, and (v) uric acid. The calculations leverage physical and chemical parameters, specific to the selected stone type, to estimate the minimum time necessary to reach a prescribed stone volume. The specific controls and mechanisms of precipitation, aggregation, and actual stone formation are not accounted for in these calculations. Thus, the choice of stone type (chemical composition) determines the default values for bulk density and chemical composition based on established reference data (see Supplementary Information, Appendix A)^18^.

To determine the time required to form a stone of prescribed type and volume, the chemical composition of the stone is used to identify the “bottleneck” urinary chemical species that act as “building blocks” in the formation of calcium oxalate, calcium phosphate, and uric acid stones. Our calculation uses highly detailed data on the chemical composition of urine (see Supplementary Information, Appendix A), which includes the specification of mmol/kidney/day and mg/kidney/day of key chemical elements in urine^19^. For a specified stone type, we identify the rate-limiting urinary solute (i.e., the constituent with the lowest urinary concentration, which thus restricts stone formation). More specifically, for the five stone types considered here, only four building blocks are analyzed to determine the rate-limiting solute for each stone, namely uric acid (C_5_H_4_N_4_O_3_), calcium (Ca^2+^), oxalate (C_2_O_4_^2−^), and phosphate (PO_4_^2−^). An example calculation is given in the Supplementary Information, Appendix A.

Calculations are made for solutions of water containing only the specific chemical species required to precipitate the different types of stones, for specific sizes of idealized spherical stones. We note that the calculations can, in the future, be modified easily to account for other stone shapes (e.g., elliptical) and combinations of stone types. We note that our calculation considers the average urine composition to be constant over the years, recognizing that urine composition is known to change constantly, being influenced by diet, genetic factors, age, gender, and environmental factors, including exposure to extreme weather and pollution. Subsequently, the stone volume is determined based on its geometric configuration (e.g., spherical) and corresponding size in millimeters (e.g., diameter), to enable calculation of the total mass of the stone (= volume × density).

By calculating the volume of urine that contains the rate-limiting solute necessary to provide an adequate “building block” material for the specified type and size of stone, and by assessing the volumetric flow rate through the kidney, one can estimate the minimum time required for stone formation. It is important to note that we assume that urine chemistry measurements reported in the literature encompass all ions present in urine, whether in dissolved or suspended particulate form, without reference to speciation. Specifically, for the case of calcium, it is assumed here that standard urine chemistry tests identify both dissolved calcium and calcium present in small, suspended calcium phosphate or calcium oxalate crystals. This is a critical assumption: if particulate or crystal forms are not included in standard urine measurements, then the amounts of building blocks available for stone formation in urine – such as reported in the Supplementary Information, Appendix A – may be significantly under-estimated, resulting in a corresponding over-estimation of the times required for stone formation.

In the context of the volumetric flow rate, we account also for another key factor that controls the rate of stone growth: a % yield is specified to account for the portion of the rate-limiting urinary solute that is captured and incorporated into the stone (in other words, an accounting of the efficiency of the stone formation process). In other words, the % yield accounts for how “efficient” the body is at forming stones as well as for how much of the urine volume actually flows past the forming stone. The % yield is affected by (*i*) the formation of multiple stones (we focus calculations on the formation of a single stone) so that a specific stone is exposed to only a fraction of the total urinary volume within the kidney; (*ii*) elimination of small crystals and stones by free flow from the kidney, which further reduces the availability of solute(s) for larger stone growth; and (*iii*) the effect of partitioning, which is the process by which movement of solutes between solution and the forming stone is limited (thus preventing excessive depletion of the solute from the urine).

The methodology developed here thus calculates an idealized minimum time for stone formation because (in addition to the assumptions described above) it is assumed – subject to the specified % yield – that *all* of the rate-limiting ions are removed from the urine prior to leaving the kidney.

## Results

### Estimated mass of stones

Table 1 shows calculations for the mass (in mg) of each of the five types of mineral precipitates, over a range of diameters (*d*, in mm), for *spherical* stones. Note that mass does not increase linearly with diameter, *d*, but according to (*d*/2)^3^ (because volume = (1/6)π*d*^3^). To illustrate the implications of the calculations presented here, a pure weddellite stone, spherical, with a diameter of 5 mm has a mass of ~ 127 mg, while a 10 mm (1 cm) stone has a mass of ~ 1000 mg (1 g). These sizes and masses correspond to measured kidney stones and kidney stone fragments^20^.

**Table 1:** Calculated mass of spherical stone types, for a range of stone diameters, *d*.

*d* (mm)	Whewellite (mg)	Weddellite (mg)	Hydroxyapatite (mg)	Brushite (mg)	Uric acid (mg)
1	1.2	1.0	1.6	1.2	1.00
3	31	27	45	33	26
4	74	65	106	77	63
5	145	127	206	151	122
6	251	219	356	261	211
10	1162	1016	1649	1210	979

### Estimated times for stone formation

Table 2 presents calculations for the formation times of various spherical stone types across a spectrum of stone diameters, *d*, assuming yield percentages of 1%, 5%, and 10% (precipitation from solution). These calculations are predicated on an average urine flow of 0.75 L/kidney/day^21,22^; this equates to ~ 250 mL/day per calyx, if the stone forms within a calyx rather than in the renal pelvis.

The choice of % yield is particularly critical, as illustrated in Table 2. The higher yield values (5%, 10%) may be regarded as exceptionally high in the context of typical geochemical scenarios, especially considering the relatively brief residence time of urine as it traverses the calyces and renal pelvis. To emphasize this point, urine production and flow rate of ~ 31 mL/kidney/h (based on 0.75 L/kidney/day), with an internal kidney void (renal pelvis) volume^23^ of up to 15 mL, leads to an average urine residence time in the kidney of only ~ 30 min.

Moreover, it is worth noting that existing literature indicates minimal to no differences between individuals who form stones and those who do not, in terms of concentrations and sizes of small calcium oxalate crystals^1^. This observation implies that variations in the concentrations of chemical components in urine are comparable to the level of measurement error. The calculated growth times (in days) shown in Table 2, based on a urine flow rate of 0.75 L/kidney/day, can be adjusted to reflect increased growth times of stones within each major calyx by multiplying all times in Table 2 by a factor of ~ 3; the volumetric flow in each of the three calyces is approximately 0.75 L/kidney/day divided by 3, resulting in an estimate of ~ 0.25 L/calyx/day). From Table 2, it is seen, for example, that formation of a spherical, 5 mm diameter weddellite stone at 1% solution yield requires 559 days to form.

**Table 2:** Calculated formation times (in days) of different spherical stone types for a range of stone diameters, *d*, assuming 1%, 5%, and 10% precipitation from solution.

*d* (mm)	Whewellite (days)			Weddellite (days)			Hydroxyapatite (days)			Brushite (days)			Uric acid (days)		
	% Solution Yield			% Solution Yield			% Solution Yield			% Solution Yield			% Solution Yield		
	1%	5%	10%	1%	5%	10%	1%	5%	10%	1%	5%	10%	1%	5%	10%
**1**	5.7	1.1	0.6	4.5	0.9	0.4	0.4	0.1	0.04	0.8	0.2	0.1	0.8	0.2	0.08
**3**	155	31	16	121	24	12	11	2.1	1.1	23	4.6	2.3	21	4.2	2.1
**4**	368	74	37	286	57	29	25	5	3	54	11	5	50	10	5
**5**	718	144	72	559	112	56	49	10	5	106	21	11	98	20	10
**6**	1240	248	124	965	193	97	85	17	9	183	37	18	169	34	17
**10**	5743	1149	574	4469	894	447	395	79	39	845	169	85	783	157	78

**Fig. 1:**
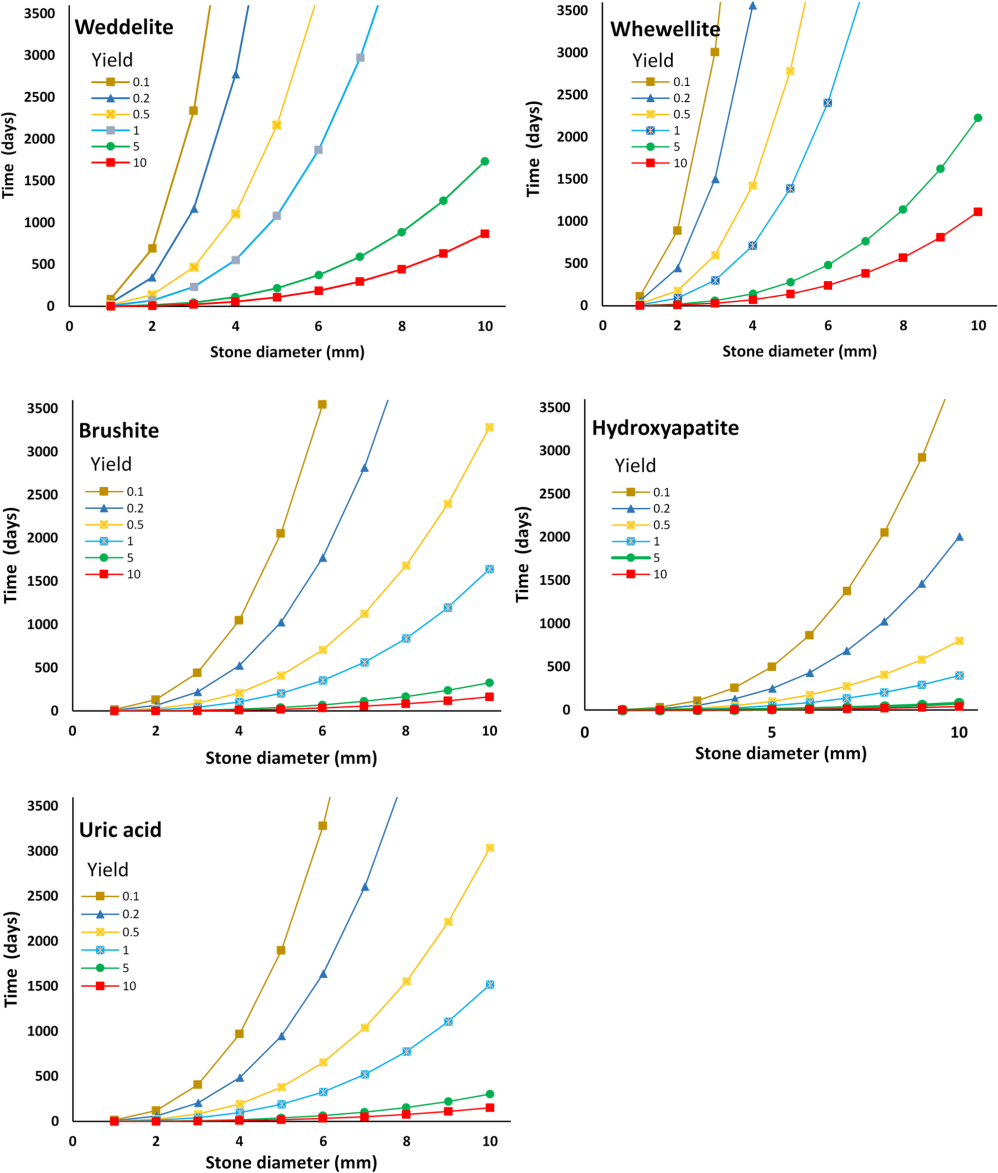
Calculated formation times for different stone types, as a function of spherical stone size up to a diameter of 10 mm. Note that times reach up to ~ 3500 days (~ 10 years). The time curves on each plot are shown for one stone with a diameter ranging from 1–10 mm, and six values (0.1%, 0.2%, 0.5%, 1%, 5%, 10%) of % yield. The % yield specifies the portion of the rate-limiting urinary solute that is captured and incorporated into the stone.

Figure 1 presents calculated formation times for different types of stones, as a function of (spherical) stone size up to a diameter of 10 mm; note that times reach up to ~ 3500 days (~ 10 years). Three additional yield percentages and multiple stone diameters are also included for comparison. Notably, minimum stone formation times increase exponentially with increasing stone diameter. Moreover, from Table 2 and Fig. 1, it is clear that the formation of a calcium oxalate stone with a diameter of 1 cm requires ~ 500 days of a constant process that consumes 10% of the limiting factor (in this case, oxalate) of the urine (and again, assuming that a stone that forms in a calyx requires a “local” yield of 30%; see explanation above).

## Discussion

Four key insights are derived from the analysis of the calculations shown above in terms of stone formation times under idealized but “definitive” conditions. We stress that the calculations are based on “idealized” conditions, as described in the Methods section, including forming a single, chemically homogeneous stone with a defined shape. The specific yield value and stone size of interest significantly influence the formation time estimates.

First, as seen in Table 2 and Fig. 1, formation times increase exponentially as stone diameters increase and % yields decrease. This behavior can be understood by recalling that as a (spherical) stone increases in diameter, *d*, the volume increases is proportional to *d*^3^; thus, increasing amounts of building block materials that are required for a stone to increase in diameter (although, on the other hand, the relative surface area available for additional deposition also increases, facilitating aggregation and stone growth. Thus, in a clinical setting, stone formation times that are faster than those predicted from the calculations shown here require higher % yields and/or a richer urine composition (e.g., unaccounted for crystals and other suspended material which may not be reported in standard urine chemistry tests, as noted above in the Methods section).

Second, the choice of % yield can give rise to stones of different composition reaching the same size. Referring to Table 2, for example, 5 mm diameter stones of hydroxyapatite and uric acid are formed at 10 days with prescribed yields of 5% and 10%, respectively; different patients are likely subject to different % yields, as a function of lifestyle, diet, metabolism, and other medical conditions. Thus, it may be speculated that either a stone-former “captures” dissolved ions and small (e.g., calcium oxalate) crystals, organic matter, and other binding agents more “efficiently” than non-stone-formers, or that a stone-former maintains higher concentrations of these components in the urine. To date, though, as noted above, the literature does not appear to report authoritative documentation of the latter case.

Third, minimum stone growth rates (based on mass balance, geometric, and physical considerations only) for each specific stone type can be deduced from the times required to reach specified stone diameters, e.g., based on Table 2 and Fig. 1 values and a prescribed % yield. These values estimate possible growth rates and suggest minimum yield values; however, it should be noted that the literature generally reports stone *presentation* in the hospital emergency room, not stone *formation*. As such, there are few authoritative, documented measurements of, e.g., formation times of new stones following operation/treatment to remove kidney stones, largely because one rarely knows for sure that the “new stone” is not a “leftover” from the operation/treatment itself. Moreover, efforts to correlate stone formation to heat waves, for example, are similarly limited by the difference between stone formation and stone presentation in the emergency room. The calculations presented here generally indicate stone growth to be sufficiently slow such that stones > 4 mm in diameter evidently cannot form (unless very high, unrealistic yield values are considered) over durations of only hours or a few days.

Furthermore, and significantly, the % yield is linked only “peripherally” to “supersaturation”. While literature often attributes stone formation to conditions of “supersaturation”, this is far from accurate. A full discussion of supersaturation appears in Supplementary Information, Appendix B. Put simply, supersaturation in pure chemical terms is of limited relevance, and does not fully explain stone formation behavior, because identification of the “*K*_sp_” of specific cations and anions (usually at room temperature, not at 37 °C) in an otherwise pure aqueous solution is not meaningful in the context of urine: urine contains multiple dissolved chemical species (cations and anion), organic components, and suspended inorganic and organic material, all of which “interfere” with determination of a “*K*_sp_” that yields a specific mineral precipitate. Moreover, as noted in the Introduction, kidney stones do not “precipitate” as a single crystal, but actually form as complex aggregates from smaller crystals and other components, processes that are not related to “supersaturation” and *K*_sp_.

Fourth, the calculation method presented here can be used as a theoretical tool, in terms of estimating how stone formation times (and growth rates) change as a function of changes in urine chemistry. For example, considering the case of urine oxalate content above the normal range, as calculated in Tables 1 and 2, and Fig. 1 (i.e., average value^19^ of 12.2 mg/kidney/d), an oxalate content above the “maximum normal” values^24^ of 40 mg/d (= 20 mg/kidney/d), say 30 mg/kidney/d (= 150% more than the average oxalate molar concentration), leads to significant differences in estimated formation time of 3 mm and 4 mm weddellite stones, for all solution yields, as detailed in Table 3. From Table 3, it is seen that even for the extreme case of a 10% yield (which translates to a 33% yield if the stone forms in a calyx rather than in the renal pelvis) and constant and very high oxalate levels, it would take at least 4.8 days to have enough “building material” to form one stone of 3 mm diameter; the formation time grows exponentially as the size of the stone increases or the yield decreases. The calculation here is based on statistical considerations, so that the occurrence of only one stone forming at any time is an unlikely scenario. If multiple stones are formed simultaneously while maintaining a steady overall yield, the formation times indicated in Table 3 and depicted in Fig. 1 must be scaled by the number of stones produced.

**Table 3:** Calculated formation times (in days) of a spherical weddellite stone, for a stone diameter of *d* = 3 mm, assuming 0.1%, 0.2%, 0.5%, 1%, 5%, and 10% precipitation from solution.

% Solution Yield	0.1%	0.2%	0.5%	1%	5%	10%
*d* = 3 mm diameter spherical weddellite stone						
“Average, normal” 12.2 mg/kidney/d urine oxalate content^19^	1207	603	241	121	60	24
“High” 30 mg/kidney/d urine oxalate content	483	241	97	48	9.7	4.8
*d* = 4 mm diameter spherical weddellite stone						
“Average, normal” 12.2 mg/kidney/d urine oxalate content^19^	2860	1430	572	286	57	29
“High” 30 mg/kidney/d urine oxalate content	1162	581	232	116	23	12
*d* = 5 mm diameter spherical weddellite stone						
“Average, normal” 12.2 mg/kidney/d urine oxalate content^19^	5586	2793	1117	559	112	56
“High” 30 mg/kidney/d urine oxalate content	2269	1135	454	227	45	23

Further to the assumptions and considerations discussed above, several additional points should be noted in the context of the theoretical model and calculations presented here. The rate of stone formation may not be constant (e.g., in terms of the calculation, the % yield may vary over time), so that, for example, as often suggested in the literature, the time for formation of an initial stone core, say from 0 to 1 mm diameter, may be considerably longer than times to further increase stone size. Thus, the model presented here may be particularly relevant only after an initial 1 mm core is established. Moreover, it should be recognized that certain locations within the kidney are more susceptible to stone formation – the lower pole calyces may contain more stones than the upper pole calyces – simply due to anatomical and gravity considerations. Finally, we stress that the presence of potential stone inhibitors, such as citrate, magnesium, and certain proteins, and the pH conditions, are not considered here. The % yield may vary as a function of the presence of these inhibitors, with inhibitors being more effective against the formation of smaller stones. Currently, a clear assessment of which biomolecules and inhibitors affect stone formation – and their quantitative impact – remains largely unknown. Future studies could certainly include these effects, but a more complete quantitative assessment of biomolecules and inhibitors is first needed. Indeed, the % yield parameter could ultimately be employed as a comprehensive adjustment factor for stone formation, allowing the incorporation of a range of considerations. This parameter has the potential to encompass biological processes, assuming appropriate estimates for these processes are available.

## Conclusions

We gain fundamental insights and define bounds on kidney stone growth rates by developing a “back to basics” analysis of urine composition and rates of kidney stone formation, based purely on mass balance, geometric, and stone density considerations. We focus here on the most common renal calculi: calcium oxalate, calcium phosphate (hydroxyapatite and brushite), and uric acid kidney stones.

By employing a purely chemical perspective to assess the volume of urine and its composition required to supply “building material” for stone formation as a function of time, without accounting for (largely unknown) complexities related to processes of small crystal aggregation and/or possible (abiotic and biotic) stone formation mechanisms, we can delineate limits on times required to form volumes of specific stone types and sizes. Overall, we find that there is an enormous difference between the presence of micron-sized crystals in solution and the formation of, e.g., 5–10 mm-sized stones. We stress that the calculations presented here are based purely on idealized, chemical considerations. The true formation rate also depends on biotic and other factors such as the presence and impact of inhibitors and biomolecules, which could either accelerate or inhibit stone formation.

The times to formation of stones of various sizes vary considerably, from hours to years, largely as a function of the amount of the “building block” material – the % yield – in urine that actually contributes to stone formation. Moreover, although we assume instantaneous formation of stone based on the amount of building material available, it remains to be determined how and at what rate building material, including micron-sized calcium oxalate, calcium phosphate, or uric acid crystals, aggregate to form a large stone. It is clear from the analysis here, however, that (*i*) stone formation is multifactorial, evidently involving other processes that could affect the stone formation rate, and that (*ii*) use of the classical chemical terminology of “the degree of supersaturation” in urine should be avoided.

This information can ultimately assist in constraining assessments of stone formation and interpretation of clinical findings.

## Electronic supplementary material

Below is the link to the electronic supplementary material.

Supplementary Material 1

## Data Availability

All data generated or analyzed during this study are included in this article [and its supplementary information files].
