# Supplementary material for: Linking basic principles of solution chemistry to kidney stone formation timelines
Source: Sci Rep. 2025 Jul 1;15:20625. doi: 10.1038/s41598-025-07193-1 (PMC12219871; doi:10.1038/s41598-025-07193-1)
Supplement: Supplementary file 1 — Supplementary Material 1 [file 41598_2025_7193_MOESM1_ESM.docx]

**Supplementary Information**

**Back to Basics: Chemical Precipitation and Potential Times to Form Kidney Stones**

**Ishai Dror^1^, Claude Merlin^1^, Yaniv Shilo^2^, Brian Berkowitz^1^**

**1. Department of Earth and Planetary Sciences, Weizmann Institute of Science, Rehovot 7610001, Israel**

2. Department of Urology, Kaplan Medical Center, Affiliated with the Hebrew University, Rehovot 7661041 **Israel**

**Appendix A: Methods – Calculation of Stone Formation**

The calculations allow for consideration of five types of stones: (*i*) calcium oxalate monohydrate (whewellite), (*ii*) calcium oxalate dihydrate (weddellite), (*iii*) hydroxyapatite, (*iv)* brushite, and (*v*) uric acid. The choice of stone type (chemical composition) determines the default values for bulk density and chemical composition based on established reference data show in Table A1 [*Walton et al., 2003*].

Table A1. Physical and chemical properties of kidney stones.

|  | Molar weight (mg/mmol) | Chemical formula | Bulk density  (g/mL) |
| --- | --- | --- | --- |
| Whewellite | 146.1123 | Ca(C_2_O_4_)•H_2_O | 2.22 |
| Weddellite | 164.1276 | Ca(C_2_O_4_)•(2.5-x)H_2_O | 1.94 |
| Hydroxyapatite | 502.31 | Ca_5_(PO_4_)_3_(OH) | 3.155 |
| Brushite | 172.0879 | Ca(PO_3_OH) •2H_2_O | 2.315 |
| Uric acid | 168.11 | C_5_H_4_N_4_O_3_ | 1.87 |

Calculations to determine the time required to form a stone of prescribed type and volume are based on the chemical composition of urine. The calculation uses highly detailed data based on established reference data shown in Table A2 [*Sarigul et al., 2019*]. It should be noted that it is not certain that standard urine tests fully measure amounts and concentrations of all key chemical building blocks (e.g., calcium, oxalate), particularly when in crystal form.

Table A2: Selected properties and chemical composition of urine (*Sarigul et al., 2019*).

| Properties and Composition | Molar Mass (g/mol) | Normal Range in humans (reference age in years) | Molarity (mmol/1.5 L) | mmol/kidney/d | mg/kidney/d |
| --- | --- | --- | --- | --- | --- |
| Volume | - | 0.8–2 L | - | - | - |
| pH | - | 4.5–8.0 | - | - | - |
| Specific gravity | - | 1.002–1.030 g/mL (all) | - | - | - |
| Osmolality | - | 150–1150 mOsm/kg (>1) | - | - | - |
| Urea (CH_4_N_2_O) | 60.06 | 10–35 g/d (all) | 249.75 | 124.9 | 7500.0 |
| Uric Acid (C_5_H_4_N_4_O_3_) | 168.11 | <750 mg/d (>16) | 1.487 | 0.7 | 125.0 |
| Creatinine (C_4_H_7_N_3_O) | 113.12 | Males: 955–2936 mg/d | 7.791 | 3.9 | 440.7 |
|  |  | Females: 601–1689 mg/d (18–83) |  | 0.0 | 0.0 |
| Citrate (C_6_H_5_O_7_^3−^) | 192.12 | 221–1191 mg/d (20–40) | 2.45 | 1.2 | 235.3 |
| Sodium (Na^+^) | 22.99 | 41–227 mmol/d (all) | 92.625 | 46.3 | 1064.7 |
| Potassium (K^+^) | 39.1 | 17–77 mmol/d (all) | 31.333 | 15.7 | 612.6 |
| Ammonium (NH_4_^+^) | 18.05 | 15–56 mmol/d (18–77) | 23.667 | 11.8 | 213.6 |
| Calcium (Ca^2+^) | 40.08 | Males:<250 mg/d | 1.663 | 0.8 | 33.3 |
|  |  | Females:<200 mg/d (18–77) |  | 0.0 | 0.0 |
| Magnesium (Mg^2+^) | 24.31 | 51–269 mg/d (18–83) | 4.389 | 2.2 | 53.3 |
| Chloride (Cl^−^) | 35.45 | 40–224 mmol/d (all) | 88 | 44.0 | 1559.8 |
| Oxalate (C_2_O_4_^2−^) | 88.02 | 0.11–0.46 mmol/d (all) | 0.277 | 0.1 | 12.2 |
| Sulphate (SO_4_^2−^) | 96.06 | 7–47 mmol/d (all) | 18 | 9.0 | 864.5 |
| Phosphate (PO_4_^2−^) | 94.97 | 20–50 mmol/d (>18) | 23.33 | 11.7 | 1107.8 |

*Example Calculation:*

An example calculation is presented for the entry in Table 2 of the main manuscript, for a spherical weddellite stone, with diameter *d =* 5 mm, and assuming 1 % solution yield, which requires ~558 days to form.

The volume of a spherical stone is (1/6)π*d* ^3^, so that choosing *d =* 5 mm (= 0.5 cm) yields a volume of 0.0654 cm^3^. The stone density is derived from the stone composition shown in Table A1. For weddellite, the stone density is 1.94 g/cm^3^. Therefore, the mass of a 5 mm diameter spherical weddellite stone, volume times density, is 0.0654 cm^3^ × 1.94 g/cm^3^ = 0.127 g = 127 mg.

The next step is to refer to the chemical formula of the stone (Table 1) and check the components that make up the stone. For weddellite, the components are calcium (Ca^2+^), oxalate (C_2_O_4_^2-^), and water. The urine composition (see Table A2) is then used to identify the limiting factor (component with the lowest molar concentration) that determines the maximum amount of stone-building material per known urine volume. For weddellite, the concentration of calcium in urine (Table A2) is 0.8 mmol/kidney/d, and for oxalate, 0.138 mmol/kidney/d. Therefore, oxalate is the limiting factor. The maximum amount of stone that can be generated per kidney per day can then be calculated, as the concentration of the oxalate (the limiting factor) is 0.138 mmol (per kidney per day) is known. Because the molar ratio of oxalate to weddellite is 1:1, the maximum amount of weddellite stone that can be formed per kidney per day is 0.138 mmol, which translates to 22.73 mg.

The % yield factor explained in the main manuscript provides a factor (or sum of factors) that indicates what portion of the maximum amount of building blocks is available for stone formation in the urine that is, in fact, consumed to build one stone in a given time. Assuming, for the example here that the yield is 1%, calculation shows that only 0.2273 mg of weddellite will be produced per day per kidney. Therefore, 558.7 days are required to produce a spherical 5 mm weddellite stone.

**Appendix B: A Discussion of "Supersaturation"**

*Basic terminology: solubility and saturation*

To explain "supersaturation" and how it is determined, it is important to understand the meaning of solubility, saturation, and some of the leading factors that affect them, as well as the difference between solution and suspension; the subject begins very simply but quickly becomes more complicated.

The term "solubility" refers to the maximum amount of a substance (solute) that can dissolve in a given solvent at a specific temperature and pressure to form a homogeneous solution. It is usually expressed in terms of the mass of solute (or number of moles) that can dissolve in a given volume of solvent to form a saturated solution. Several factors influence solubility:

1. Nature of the solute and solvent: Polar solutes (like phenol, acids, creatinine, hippuric acid, citric acid and many ions like Na^+^, K^+^, Ca^2+^ and Cl^-^) tend to dissolve more easily in polar solvents (like H_2_O or methanol), while nonpolar solutes (including aliphatic and aromatic hydrocarbons, such as cymenes, undecane, and himachalane that are found in urine [*Bouatra et al., 2013*]) dissolve better in nonpolar solvents (e.g., toluene or hexane).
2. Temperature: In general, the solubility of most solid solutes in liquid solvents increases with an increase in temperature.
3. Pressure: For most solids and liquids, changes in pressure have little effect on solubility. However, for gases dissolved in liquids, an increase in pressure generally increases solubility.

The maximum solubility of a solute in a solvent is reached when the solution is saturated, meaning that any additional solute added will not dissolve and will accumulate as a solid phase. The concentration of the solute in a saturated solution is known as the solubility limit.

The term "saturation" refers to a state in which a solution contains the maximum possible concentration of a solute under specific conditions. The saturation point is influenced by factors such as temperature, pressure, and solution composition.

*K_sp_:* *Quantifying the equilibrium “tango” of ions and solids*

In the intricate dynamics of aqueous equilibria, the solubility product constant, *K*_sp_, plays a crucial role in governing the harmonious interplay between ionic solids and their constituent ions in solution. This dimensionless quantity, calculated as the product of ionic activity coefficients raised to their stoichiometric coefficients in the dissolution equation, provides a succinct measure of the intrinsic tendency of a compound to populate the liquid phase.

The equilibrium established between the dissolving solid and its liberated ions, a dynamic interplay governed by Le Chatelier's principle, defines the fundamental principle underlying *K*_sp_. The rate of dissolution precisely balances the rate of recrystallization, maintaining a constant concentration of solvated ions. A higher *K*_sp_ value signifies a greater tendency for the constituent ions to be in a dissolved form. Consider, for instance, the contrasting fates of silver chloride (AgCl) and barium sulfate (BaSO₄). With a relatively high *K*_sp_, AgCl readily relinquishes its silver and chloride ions, populating the aqueous phase and yielding a saturated solution. Conversely, the significantly lower *K*_sp_ of BaSO₄ dictates a more restrained participation in the aqueous solution, resulting in a low concentration of ions and a predominantly solid existence.

Understanding *K*_sp_ transcends mere prediction of solubility. It empowers chemists to delve into the intricate world of precipitation reactions, predict the formation of precipitates under varying conditions, and even tailor materials with tailored solubilities for diverse applications. As the thermal energy of the system increases, so too does the freedom of movement for the ions, often manifesting as an elevation in *K*_sp_ and, consequently, enhanced solubility.

The above explanation of *K*_sp_ applies to a specific (and "simple") solution, often referring to pure water and one type of salt at a specific temperature. However, urine is a very complex suspension (see explanation below for the difference between solution and suspension), with numerous components that change continuously, thus making the *K*_sp_ values rough estimates at best and irrelevant in most cases.

*More terminology: solutions and suspensions*

At this point, it is important to focus on the difference between "solutions" and "suspensions". Solutions and suspensions are two types of mixtures with distinct, differentiating characteristics.

"Solutions" are homogeneous mixtures, meaning that the solute is distributed uniformly throughout the solvent. The components are mixed at the molecular or ionic level, resulting in a single-phase system. In solutions, the particles (ions or molecules) of the solute are very small, typically less than 1 nm in diameter. Solutions are usually transparent and do not exhibit the Tyndall effect (scattering of light), making them difficult to see. The solute does not settle at the bottom of the container over time, but remains evenly dispersed in the solvent.

"Suspensions" are heterogeneous mixtures, meaning that the particles of the solute are not distributed uniformly throughout the solvent. They form two distinct phases. Suspensions contain larger particles, often visible to the naked eye. These particles can be larger than 1 µm in diameter. Suspensions are often cloudy or opaque due to the presence of larger particles, and they exhibit the Tyndall effect, causing light to scatter. The particles in a suspension tend to settle at the bottom of a container over time due to gravity; agitation or stirring is required to maintain uniformity.

In summary, the key difference between *solutions* and *suspensions* lies in the homogeneity of the mixture. Solutions are homogeneous, with small particles that do not settle, while suspensions are heterogeneous, with larger particles that may settle over time.

*Supersaturation*

"Supersaturation" refers to a state in which a solution contains a higher concentration of a dissolved substance than would normally be possible under specific conditions. In an unsaturated solution, the concentration of the solute is below its saturation point, and in a saturated solution, it has reached the maximum concentration at a given temperature and pressure. However, in a supersaturated solution, the concentration exceeds the saturation point without the solute precipitating out.

Supersaturation typically occurs when a solution is prepared at an elevated temperature and then slowly cooled, preventing the solute from precipitating out immediately. This can result in a solution that temporarily holds more dissolved substance than it would under normal conditions. Supersaturation is often an unstable state, and the excess solute may precipitate out if the conditions change or if a seed crystal or foreign particle is introduced, providing a surface for crystal formation.

When the studied solution comprises multiple components, one must consider potential interactions and feedback mechanisms among the various species. For example, common ions such as, e.g., Ca^2+^ (found in calcium phosphates and calcium oxalates), or CO_3_^2-^ (that can react with many cations like Ca, Mg, and Fe). The "common ion effect" is a phenomenon observed in solutions when the addition of a salt containing an ion that is already present in the solution causes a reduction in the solubility of another salt. This effect is based on Le Chatelier's principle, which states that if a system at equilibrium is disturbed, the system will adjust itself to counteract the disturbance.

In the context of the common ion effect, consider a solution in which a poorly soluble salt is dissolved. This salt will undergo dissociation into its constituent ions (e.g., NaCl dissociates to Na^+^ and Cl^-^). If a second salt (e.g., NaBr or KCl) is added to the solution that contains an ion already present in the first salt, the common ion effect is at work.

The common ion effect can be explained using the following general equilibrium equation for a poorly soluble salt: M*_a_*​X*_b_*​_(_*_s_*_)_ ⇌ *a*M*^n^*^+^_(_*_aq_*_)_ + *b*X*^m^*^−^_(_*_aq_*_)_. Here, M*_a_*X*^b^* is the poorly soluble salt, and it partially dissociates into its constituent ions M^n+^ and X^m−^. Now, if a salt containing either M^n+^ or X^m−^ is added to the solution, it provides additional amounts of these ions. According to Le Chatelier's principle, the equilibrium will shift to the left to counteract this increase in the concentration of the common ion. As a result, the dissociation of the poorly soluble salt is reduced, leading to a decrease in its solubility.

The common ion effect is particularly relevant in the context of precipitation reactions and the solubility of salts in solution. It can be used to predict and manipulate the solubility of salts by considering the presence of common ions in the solution.

*Supersaturation in the context of kidney stone formation*

Based on the explanation above and observations, it must be recognized that:

(*i*) Urine is a suspension comprising a large variety of undissolved particles [*e.g., Elliot and Rabinowitz, 1980; Sayer et al., 2004; Lee et al., 2022*], as well as dissolved components.

(*ii*) The composition of urine is complex and constantly changes therefore multiple “common ion” effects and feedback mechanisms are expected, which render supersaturation calculations as complicated and unstable.

(*iii*) The concentration of elements like calcium or magnesium in urine is affected by a combination of dissolved ions, (organic) complexes that comprise these elements, and suspended particles (mineral and composite materials). Therefore, to accurately measure specific ion concentrations, the analysis method must consider the complicated composition of urine. The information "hidden" in the forms a given element is found (including the type of complexes and size, size distribution, bulk composition, and morphology of particles) could be a key to early detection and understanding of stone formation. Conventional (clinical) urine testing is used to detect various dissolved and suspended particulate matter, with a focus on diagnosis and monitoring of conditions such as infections, kidney disease, diabetes, dehydration, and metabolic disorders. The key measurements include:

- Dissolved substances, generally characterized by pH and specific gravity, and detected through chemical analysis: electrolytes (e.g., sodium, potassium, chloride, and bicarbonate), metabolites (e.g., urea, creatinine, uric acid, and other waste products), glucose, proteins, and ketones.
- Suspended particulates and crystals, generally detected through microscopic analysis: cells (red blood cells, white blood cells, epithelial cells), crystals (e.g., calcium oxalate, uric acid, cystine), casts (structures formed in the kidney tubules, composed of cells, proteins, or fat), bacteria, and yeasts or parasites.

(*iv*) The temperature and pressure in the kidney are relatively stable, so that the likelihood that these factors generate supersaturation conditions (as occurs in other research fields) is very low.

(*v*) Even if small crystals are formed by precipitation of supersaturated components found in the urine, all laboratory experiments to date, even in much more extreme conditions, lead to the formation of mineral particles with sizes that do not exceed 100 µm [e.g., *Hennequin et al., 1993; Grases et al., 1996; Grases et al., 2000; Vaitheeswari et al., 2015; Carino et al., 2018*]. These small particles are expected to be flushed from the kidney.

**References**

Bouatra, S. *et al.* The human urine metabolome. *PLOS ONE* **8**, e73076. <https://doi.org/10.1371/journal.pone.0073076> (2013).

Carino, A. *et al.* Formation and transformation of calcium phosphate phases under biologically relevant conditions: Experiments and modelling. *Acta Biomater.* **74**, 478-488. <https://doi.org/10.1016/j.actbio.2018.05.027> (2018).

Elliot, J. S. & Rabinowitz, [I. N.](https://www.auajournals.org/doi/10.1016/S0022-5347%2817%2955918-2) Calcium oxalate crystalluria: crystal size in urine. [*J. Urol.*](https://www.auajournals.org/journal/juro) **123**, 324–327. <https://doi.org/10.1016/S0022-5347(17)55918-2> (1980).

Grases, F. *et al.*Phosphates precipitating from artificial urine and fine structure of phosphate renal calculi, *Clin. Chim. Acta* **244**, 45–67. <https://doi.org/10.1016/0009-8981(95)06179-7> (1996).

Grases, F. *et al.* Uric acid calculi: types, etiology and mechanisms of formation, *Clin. Chim. Acta* **302**, 89–104. <https://doi.org/10.1016/S0009-8981(00)00359-4> (2000).

Hennequin, C. *et al.* A new approach to studying inhibitors of calcium oxalate crystal growth. *Urol. Res.* **21**, 101-108. <https://doi.org/10.1007/BF01788827> (1993).

Lee, A.J. *et al.*Differential identification of urine crystals with morphologic characteristics and solubility test. *J. Clin. Lab. Anal*. **36**, e24707. doi:10.1002/jcla.24707 <https://onlinelibrary.wiley.com/doi/10.1002/jcla.24707> (2022).

Sarigul, N., Korkmaz, F. & Kurultak, İ. A new artificial urine protocol to better imitate human urine. *Sci. Rep.* **9**, 20159. <https://doi.org/10.1038/s41598-019-56693-4> (2019).

Sayer, J. A., Carr, G. & Simmons, N. L. Calcium phosphate and calcium oxalate crystal handling is dependent upon CLC-5 expression in mouse collecting duct cells. [*Biochim. Biophys. Acta – Mol. Basis Dis.*](https://www.sciencedirect.com/journal/biochimica-et-biophysica-acta-bba-molecular-basis-of-disease) **1689**, 83–90. <https://doi.org/10.1016/j.bbadis.2004.02.007> (2004).

Vaitheeswari, S. *et al.*Studying inhibition of calcium oxalate stone formation: an in vitro approach for screening hydrogen sulfide and its metabolites, *Int. Braz. J. Urol.* **41**, <https://doi.org/10.1590/S1677-5538.IBJU.2014.0193> (2015).

Walton, R. C., Kavanagh, J. P. & Heywood, B. R. The density and protein content of calcium oxalate crystals precipitated from human urine: a tool to investigate ultrastructure and the fractional volume occupied by organic matrix, *J. Struct. Biol.* **143**, 14-23.
 <https://doi.org/10.1016/S1047-8477(03)00117-5> (2003).
